# Melatonin rhythms beyond the pineal organ: gene expression of receptors and biosynthesis enzymes in wild and F1 Senegalese sole

**DOI:** 10.1007/s10695-025-01502-1

**Published:** 2025-05-01

**Authors:** Francisca Félix, Catarina C. V. Oliveira, Ignacio Martín, Manuel Manchado, Francisco J. Sánchez-Vázquez, Elsa Cabrita, Luisa M. Vera

**Affiliations:** 1 Centre of Marine Sciences, CCMAR/CIMAR-LA), University of Algarve, Gambelas Campus, 8005-139 Faro, Portugal; 2 Department of Physiology, Faculty of Biology, Regional Campus of International Excellence “Campus Mare Nostrum”, University of Murcia, 30100 Murcia, Spain; 3 Spanish Institute of Oceanography, Santander Oceanographic Centre. Promontorio de San Martín, S/N. Apdo 240, Santander, 39080 Spain; 4 Junta de Andalucia, Centro IFAPA, El Toruño, Cádiz, Spain

**Keywords:** *Solea senegalensis*, Melatonin receptors, Extra-pineal production, Rt-PCR

## Abstract

Fish gonadal melatonin production is still unexplored and could contribute to a better understanding of its role in reproduction control, especially for species with reproductive impairments. This study aimed to comprehend if Senegalese sole testes are an extra-pineal production site of melatonin and if it has seasonal and daily variations. Wild and F1 broodstocks were sampled in the breeding season (BS) and out of the reproductive season (OS), at mid-light (ML) and mid-dark (MD) daytimes. Blood plasma melatonin concentration was determined by radioimmunoassay (RIA). The expression of genes involved in melatonin biosynthesis (*tph1a*, *tph2*, *hiomt1*, *aanat1a*, *aanat1b*, and *aanat2*) and melatonin receptors (*mel1*, *mel1c*, and *mel2*) was evaluated in the brain, eye, and testis by quantitative real-time PCR (qPCR). Plasma melatonin concentration in wild sole displayed day/night differences in both seasons (average ML: 36 ± 22 pg/mL, MD: 108 ± 63 pg/mL), whereas differences in the F1 broodstock were only found OS (ML: 100 ± 54 pg/mL, MD: 187 ± 88 pg/mL). Gene expression of *mel1* and *mel2* receptors, and *tph1a*, *aanat1a*, *aanat2*, and *hiomt1* enzymes was detected and quantified in the fish testes. Moreover, daily and seasonal fluctuations in the expression of those genes were found in all tissues and broodstock groups. However, the F1 group showed distinct gene expression patterns compared to the wild type, suggesting a disruption in the circadian system. This study revealed that Senegalese sole testes are a melatonin production site and, at the same time, suggested a dysregulation in the hypothalamus-pituitary-gonad (HPG) axis of F1 males.

## Introduction

Research on circannual and circadian melatonin production in peripheral tissues has been a hot topic in chronobiology, especially after earlier studies documented that mitochondria maintain melatonin concentrations independent of blood levels (Reiter et al. 2017). Analytical techniques such as radioimmunoassay (RIA) or ELISA have been used to detect and quantify melatonin in peripheral tissues of several fish species, such as the eye of European seabass (*Dicentrarchus labrax*) (García‐Allegue et al., 2001), zebrafish (*Danio rerio*) (Cahill 1996), goldfish (*Carassius auratus*) (Iigo et al. 1997), and brook trout (*Salvelinus fontinalis*) (Zachmann et al. 1992). Also, melatonin has been found and measured in gastrointestinal tissues, where concentrations are even higher than in the bloodstream (Bubenik and Pang 1997; Vera et al. 2007). These studies sparked the search for other extra pineal production sites and, despite the important role of this clock hormone in the regulation of reproduction (Falcón et al. 2007), melatonin production in fish gonads has not been properly investigated until now. Recently, our group has reported that melatonin is present in fish seminal plasma, where it displays day/night differences, as it does in blood plasma, although the correlation between seminal and blood plasma levels seems to be species-specific (Félix et al. 2023). Moreover, melatonin contributed to the antioxidant status of seminal plasma, similar to the gonadal antioxidant protection previously advanced by other authors (Maitra and Hasan 2016). Some experiments in carp (*Catla catla*) have also pointed to an important role of melatonin in annual testicular (Bhattacharya et al. 2007) and oocyte (Mondal et al. 2017) maturation, although the first evidence of gonadal melatonin production was found in zebrafish testis, where the expression of key enzymes involved in melatonin synthesis (*tph1*, *aanat1*, *aanat2*, and *hiomt*) has been determined (Devi et al. 2021).

The biosynthesis of this indolamine relies on the bioavailability of tryptophan and a set of enzymes necessary to transform it into melatonin (Zhao et al. 2019). In animals, this process starts with the action of tryptophan hydroxylase (TPH), which converts tryptophan into 5-hydroxytryptophan, which is then decarboxylated by aromatic amino acid decarboxylase (AADC), forming serotonin. Afterwards, two more enzymatic processes occur: Serotonin is catalyzed by arylalkylamine N-acetyltransferase (AANAT), originating N-acetylserotonin and, finally, the hydroxyindole-O-methyltransferase (HIOMT) converts the latest into melatonin (Tan et al. 2016). It is worth mentioning that recent experiments with European flounder (*Platichthys flesus*) skin tissue suggest the existence of another pathway for melatonin biosynthesis in which 5-hydroxytryptamine (5-HT) is first methylated into 5-methoxytryptamine (5-MT), which is then acetylated to melatonin (Gozdowska et al. 2024), but such process was not found to be described in other tissues or fish species yet.

Unlike other vertebrates, teleost fish possess two AANAT genes (AANAT1, AANAT2), which expression levels differ between tissues. In addition, in some species such as Senegalese sole (*Solea senegalensis*), there are AANAT1a and AANAT1b subtypes (Isorna et al. 2011). The presence of these enzymes in peripheral organs indicates melatonin production in extra pineal sites, as it was already demonstrated in several tissues of rainbow trout (*Oncorhynchus mykiss*) (Fernandez-Duran et al. 2007; Muñoz-Perez et al. 2016), in the retina of brown trout (Besseau et al. 2006), in carp gut (Devi et al. 2016), and zebrafish testis (Devi et al. 2021), as previously mentioned. Moreover, likewise in the pineal organ, melatonin production in other tissues also displays daily and seasonal rhythmicity, probably caused by rhythms in the expression of the enzymes involved in its biosynthesis (Isorna et al. 2009; Falcón et al. 2010; Maitra and Pal 2017). Adult Senegalese sole present high expression levels of AANAT1 in the retina (with subtypes displaying different rhythms) whereas AANAT2 is more associated with pineal functions (Klein 2007; Lan-Chow-Wing et al. 2014), including the regulation of rhythmic physiological processes such as reproduction (Reiter et al. 2009). In fact, melatonin is responsible for transducing the photoperiodic information into the animal body, triggering the hormonal cascade responsible for gonadal maturation and sperm production (Chaube et al. 2020). Senegalese sole presents reproductive impairment when fish are born and raised in captivity (first generation—F1), which is characterized by the absence of courtship behavior, lack of natural spawning, overall poor gamete quality, and low sperm volume (Cabrita et al. 2011; Fatsini et al. 2020). In this species, melatonin receptors have been identified in several organs (Confente et al. 2010), but the gonads have not been explored yet. Also, both seminal plasma melatonin (Félix et al. 2023) and sperm motility (Félix et al. 2024) are higher at night, but whether melatonin is produced by the testis itself remains unknown.

The way melatonin reaches and acts on the biological targets is still unclear, as several mediated and non-mediated transport mechanisms have been observed, as well as paracrine and autocrine actions (Mayo et al. 2018). In fish, three melatonin receptors have been described (MT1, MT2, and MT3), although their presence varies between tissues and species (Falcón et al. 2010). Thus, the expression of melatonin receptors has been determined in many organs from marine and freshwater species, including brain, eye, muscle, heart, intestine, liver, kidney, gills and skin (Park et al. 2007; Sauzet et al. 2008; Ikegami et al. 2009; Confente et al. 2010; Ciani et al. 2019; Zhao et al. 2021). Regarding the gonads, several studies have reported the presence of melatonin receptors in fish ovary (Chattoraj et al. 2009; Moniruzzaman and Maitra 2012; Lombardo et al. 2014). However, information about melatonin receptors in testis is scarce (Zhao et al. 2021). Therefore, this study aimed to determine if melatonin is produced by gonads in male Senegalese sole broodstock (wild and F1). Also, seasonal (breeding and out of reproductive season) and daily (mid-light and mid-darkness) variations in melatonin levels, in the expression of genes involved in its biosynthesis and melatonin receptors were investigated within each broodstock, in three target organs: brain, eye, and testis.

## Material and methods

### Experimental design

In this experiment, wild and F1 Senegalese sole (*Solea senegalensis*) males with mean body weight (BW) of 607 ± 184 g were sampled in and out of the reproductive season, at both mid-light (ML) and mid-dark (MD) daytimes, for collection of blood plasma, gonads, brain, and eye samples. Wild animals were caught from the extensive fish farm Aqualvor (Portimão, Portugal), in both seasons and maintained at *Ramalhete* station, the experimental facilities of the University of Algarve, under natural photoperiod and controlled temperature. After 1 month of acclimatization, in the breeding season (BS), 10 wild male fish were sampled, with the photoperiod being 12 h:12 h light:dark (12L:12D), and water temperature 20.4 °C (ML; *n* = 5) and 19.6 °C (MD; *n* = 5). Out of reproductive season (OS), nine wild males were sampled, which were exposed to a photoperiod of 10L:14D and water temperature of 15.8 °C (ML; *n* = 4) and 13.2 °C (MD; *n* = 5). The F1 males used in BS were from an established batch from IFAPA *El Toruño* (Cádiz, Spain), wherein the photoperiod was 14L:10D and water temperature 21.2 °C (ML; *n* = 7) and 20.5 °C (MD; *n* = 8). The F1 males used at OS were from an established batch from IEO (Santander, Spain), wherein the photoperiod was 16L:8D and water temperature 12.8 °C (ML; *n* = 5) and 12.2 °C (MD; *n* = 7).

For each fish stock (Wild/F1), four experimental subgroups were made: BS-ML, BS-MD, OS-ML, and OS-MD. At each sampling point, fish were euthanized with an overdose of anesthesia (1000 ppm phenoxyethanol). Blood was collected from the caudal vein with 1 mL heparinized syringe, immediately centrifuged, and plasma stored at − 80 °C until further analysis. Fish were then dissected to collect brain, eye, and gonad samples. All tissues were plunged directly into liquid nitrogen and stored at − 80 °C until RNA extraction.

### Blood melatonin determination

Melatonin quantification was performed by radioimmunoassay (RIA) using the Melatonin RIA kit (RE 29301, IBL International, Germany), following the manufacturer protocol. Previously, this kit had been used and validated for melatonin detection in fish blood plasma by our group (Félix et al. 2023). For the analysis, 100 µL of each sample was used and the standard curve was prepared. The enzyme solution was added, and samples were centrifuged and incubated for 2 h at 37 °C. After, the assay buffer, I^125^ melatonin and antiserum were added to samples and centrifuged, followed by an overnight incubation at room temperature. On the second day, samples were incubated with the precipitating reagent, centrifuged, and the supernatant aspirated manually with a Pasteur pipette. Finally, radioactivity was measured for 1 min on an automatic gamma counter (WALLAC 1470, Perkin Elmer, Waltham, MA, USA).

### RNA extraction and cDNA synthesis

The brain, eye, and gonad tissue samples were homogenized in 1 mL of TRI Reagent Solution (Invitrogen, Lithuania) using a TissueLyzer LT bead mill (Qiagen Ltd. Manchester, UK). Total RNA was extracted following the manufacturer’s instructions. RNA pellets were resuspended in DNase RNase-free distilled water (Merck Millipore). The total RNA concentrations were measured in a Nanodrop 2000 spectrophotometer (Thermo Fischer Scientific, WA, USA). cDNA synthesis was performed from 1 μg of total RNA, using the QuantiTect Reverse Transcription kit (Qiagen Ltd. Manchester, UK). The resulting cDNA was diluted tenfold with DNase RNase-free distilled water (Merck Millipore).

### Quantitative real-time PCR

The relative expression of nine selected genes was determined (*n* = 6 samples/experimental subgroup): three melatonin receptors genes (*mel1*, *mel1c*, and *mel2*), and five other genes involved in melatonin synthesis (*tph1a*, *tph2*, *hiomt1*, *aanat1a*, *aanat1b*, and *aanat2*). As reference genes, *18S*, *ubq*, *β-actin*, and *rpl13* were employed (Table 1).

**Table 1:** Sequences of the primers used for the quantitative real-time PCR (RT-PCR). Temperature of annealing was 60 ⁰C for each primer. FW, forward; RV, reverse

Gene	Accession number	Amplicon size (bp)	Primer FW (5′–3′)	Primer RV (5′ to 3′)
*18 s*	EF126042.1	148	GGCCGTTCTTAGTTGGTGGA	TTGCTCAATCTCGTGTGGCT
*rpl13*	AB374948.1	103	AGTCTCTTCAGGCCAACGTG	TCCTCGGTTCCATCTCCCTT
*bact*	DQ485686.1	91	TGTGCGGGTATCATTTGCCT	GTGCGGCGATTTCATCTTCC
*ubq*	AB374981.1	156	CTCAGACAGCTGGCCCAGAA	CAGCCCTCGCGTCTACTTCA
*ef1a*	DQ788621.1	103	ACAACGTGGGCTTCAACATC	AAACTTTCAGCAGCCTTGGG
*mel1*	FM213463.1	139	CTTCAACAGCTGCCTCAACG	CCGTTTGTCCTCGTCCGTTA
*mel2*	FM213464.1	115	TAATCTGGCTGCTCACCGTC	GTGTAGGAGCTGCTGACGTT
*aanat1a*	GQ340971.1	73	GGGACGTCTGGTGGCTTTTA	GAGAGTCAGAGCGTCCATGG
*aanat2*	GQ340973.1	82	GTTCGAGGAGGGACAACTGG	CTGAGTCATGGCCTCCTGTG
*tph1a*	XM_044037153.1	79	AACAAGAAGGGAAGCTGCGA	TTTCCGGAGAGTGCATGCTT
*hiomt1*	FN432777.1	53	GATGTGGTCAGGGCCTTTGA	CCGAGGTCACAGATGGCTTT

QuantStudioTM 5 Flex (Thermo Fisher Scientific, Waltham, USA) programmed to perform the following protocol: UDG pre-treatment at 50 °C for 2 min preceding thermal cycling, which was initiated at 95 °C for 10 min, followed by 40 cycles with a denaturing step at 95 °C for 15 s and annealing for 60 s at 60 °C. The amplification cycle was followed by a temperature ramp with 0.1 °C increments ranging between 60 and 95 °C for the melt-curve analysis to verify that no primer-dimer artefacts were present and each qPCR assay only generated one product. qPCR was carried out in 96-well plates as duplicates per sample. The final PCR reaction volume was 10 μL:2.5 μL of cDNA, 5 μL of the qPCR Master Mix, and 2.5 μL of the forward and reverse primers (400 nM of each). Reactions included systematic negative controls containing no cDNA (NTC, no template control) and a set of reactions containing serial dilutions of all the pooled cDNA experimental samples to calculate qPCR efficiencies. For each gene, the efficiency of primers was previously verified and validated using the same cDNA serial dilutions, ranging from 91 to 104%. The new primers employed in this study were designed with the PRIMER3 Software (Untergasser et al., 2012), and target specificity was checked in silico using Primer-Blast (NCBI).

### Data analysis

The relative expression of the target genes in all tissues was calculated by the ΔΔCt method (Pfaffl 2001), where the most stable reference genes were utilized for each organ according to RefFinder (Xie et al. 2012): *18S*, *ubq*, and *rpl13* (gonad and brain); *18S*, *ubq*, and *β-actin* (eye).

For each sole stock (Wild/F1), any statistical differences in gene expression were analyzed by a two-way ANOVA (ANOVA II), which tested the effects of the fixed factors “season” (BS, OS) and “time” (ML, MD), as well as the “season × time” interaction, followed by post hoc Tukey pairwise comparisons when the “treatment × time” interaction was significant. All the statistical analyses were conducted using the open-source software R (R Core Team, R Foundation for Statistical Computing). The significance level was set at *p* < 0.05. Data representation was done using the software GraphPad Prism 9.

### Ethics statement

All experimental procedures were conducted in accordance with ARRIVE guidelines, with directives 86/609/EU and 2010/63/EU of the European Parliament and Council, and Portuguese legislation for the use of laboratory animals (PORT 1005/92). The experimental procedures were previously approved by the Portuguese direction for veterinary and food services (DGAV) (license number 003289), and it is certified that the animals involved in the present study were maintained and handled according to the best practices.

## Results

### Blood melatonin levels

**Fig. 1:**
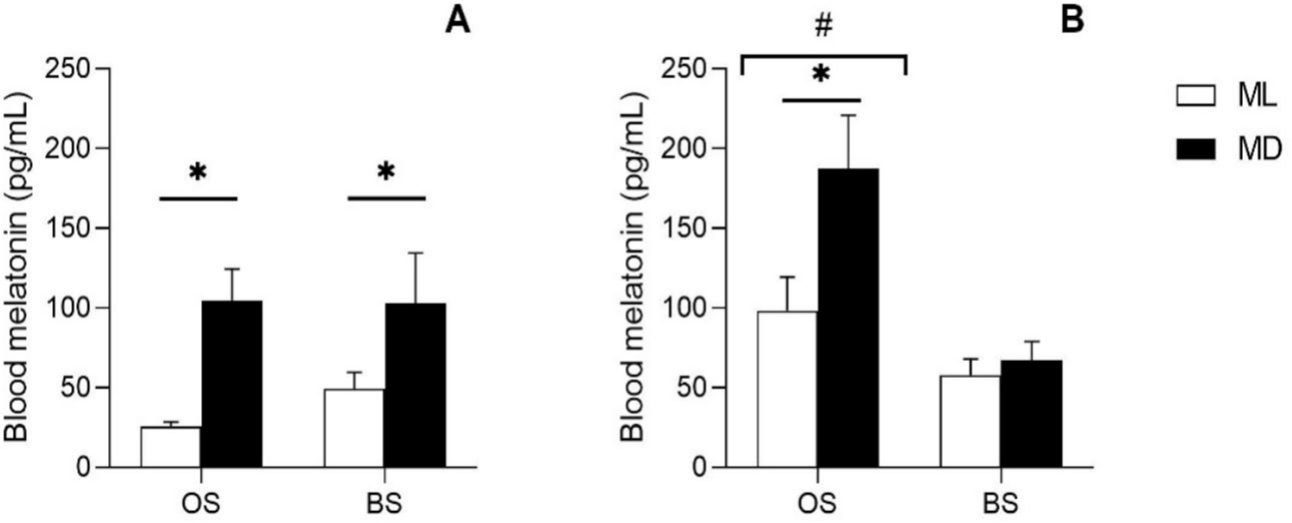
ML and MD melatonin concentration in wild (**A**) and F1 (**B**) Senegalese sole blood plasma at breeding season (BS) (wild: *n* = 5; F1: ML *n* = 7, MD *n* = 8) and out-of-season (OS) (wild: ML *n* = 4, MD *n* = 5; F1: ML *n* = 5, MD *n* = 7). Data are expressed as mean ± SE. ANOVA II significant differences between ML and MD are identified with an asterisk and between seasons with a cardinal (#). Statistical differences were considered when *p* < 0.05

The results from RIA analysis revealed that daytime (ML, MD) had a significant effect (ANOVA II, *p* < 0.05) on the blood melatonin concentration, showing higher values at MD irrespective of the broodstock origin (Fig. 1). In addition, in F1 males, season had a strong effect (ANOVA II, *p* < 0.001) on melatonin concentration, being higher at OS than at BS, irrespective of the daytime (Fig. 1a). In wild sole, significant day/night differences in melatonin levels were found at both OS and BS (average ML: 36 ± 22 pg/mL, MD: 108 ± 63 pg/mL) (Fig. 1b). However, in F1 males, such difference was only observed at OS (ML: 100 ± 54 pg/mL, MD: 187 ± 88 pg/mL) (Fig. 1a).

### Gene expression

#### Brain

**Fig. 2:**
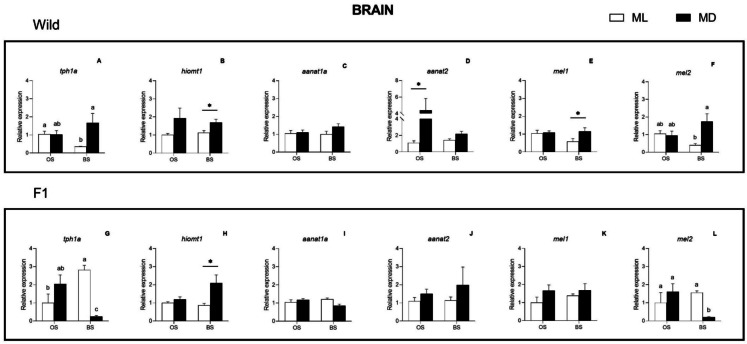
Seasonal and light–dark changes in the relative expression of *tph1a*, *hiomt1*, *aanat1a*, *aanat2*, *mel1* and *mel2* in wild (**A**–**F**) and F1 (**G**–**L**) Senegalese sole brain. Results are represented as mean ± SE. Student-*t* test significant differences between ML and MD are identified with an asterisk; ANOVA II differences are identified with different letters. Statistical differences were considered when *p* < 0.05

In brain, two-way ANOVA revealed a significant interaction between factors (season × daytime, *p* < 0.01) for *tph1a* and *mel2* in both sole broodstocks (Fig. 2a, f, g, l) and for *aanat1a* in F1 males (Fig. 2i). Significant day/night differences were observed in the expression of *tph1a* and *mel2* during the BS for both wild and F1 fish, although opposite patterns were found: i.e., in wild males, the expression levels were higher at MD (Fig. 2 a and f) whereas in the F1 group higher expression was measured at ML (Fig. 2 g and l). A significant main effect of daytime was observed for *hiomt1* expression in both broodstocks (Fig. 2 b and h) and for *aaant2* and *mel1* in wild males (Fig. 2 d and e) (ANOVA II, *p* < 0.01). In all cases, gene expression was higher at MD than at ML. Moreover, further analysis revealed ML/MD significant differences during the BS for *hiomt1* (in both broodstocks) and *mel1* (in wild males) (Student-*t* test, *p* < 0.05) (Fig. 2 b, e, h). Also, the *annat2* gene showed higher expression at MD in wild males, but only at OS (Fig. 2d).

#### Eye

**Fig. 3:**
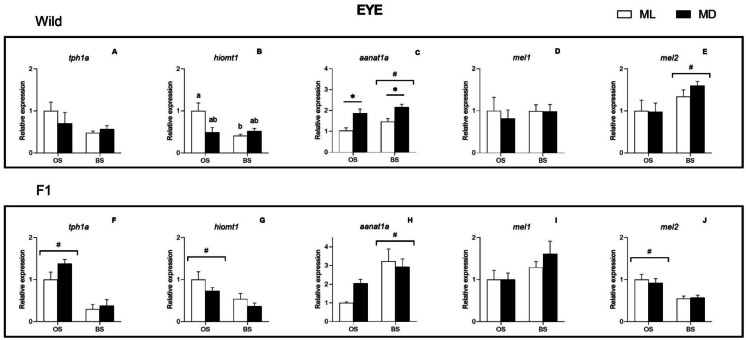
Seasonal and light–dark changes in the relative expression of *tph1a*, *hiomt1*, *aanat1a*, *mel1* and *mel2* in wild (**A**–**E**) and F1 (**F**–**J**) Senegalese sole eye. Results are represented as mean ± SE. Student-*t* test significant differences between ML and MD are identified with an asterisk; ANOVA II differences are identified with different letters and with cardinal (#) between seasons. Statistical differences were considered when *p* < 0.05

The ANOVA II revealed a significant interaction between season and daytime for *hiomt1* expression in wild males, being downregulated at ML during BS (ANOVA II, *p* < 0.05) (Fig. 3b). Moreover, a significant effect of season was found on the expression of *tph1a*, *hiomt1*, *aanat1a* in F1 males (Fig. 3f–h) and of *mel2* in both broodstocks (Fig. 3e and j) (ANOVA II, *p* < 0.05). Thus, in F1 males, the expression of *tph1a*, *hiomt1*, and *mel2* was higher at OS, whereas *annat1a* and *mel2* presented higher expression during BS in both broodstocks and wild males, respectively. In addition, *aanat1a* expression in wild males was also impacted by daytime, being more expressed at MD than at ML, irrespective of the season (ANOVA II, *p* < 0.001) (Fig. 3c). Differently from the other two tissues, the gene *aanat2* did not express on the Senegalese sole eye, regardless of the broodstock.

#### Gonad

**Fig. 4:**
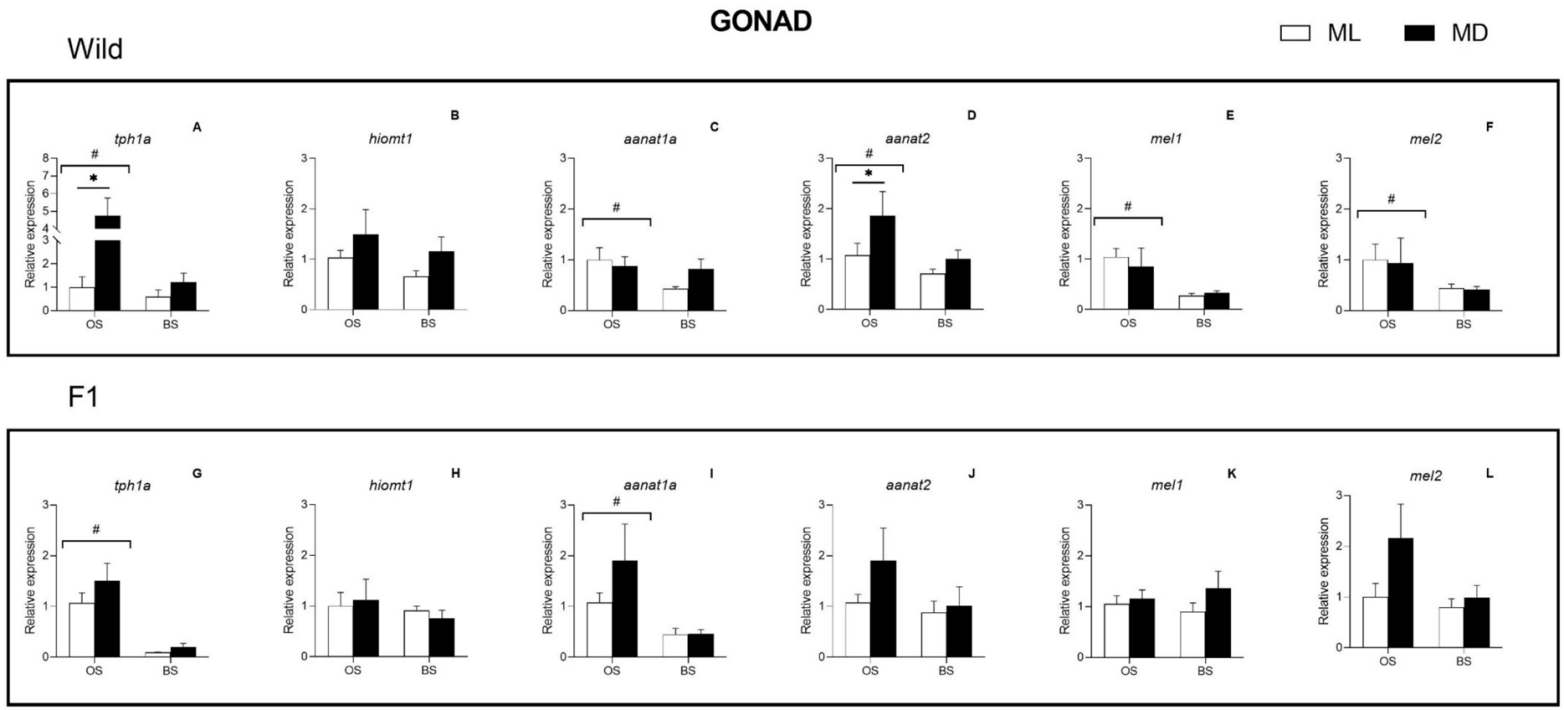
Seasonal and light–dark changes in the relative expression of *tph1a*, *hiomt1*, *aanat1a*, *aanat2*, *mel1* and *mel2* in wild (**A**–**F**) and F1 (**G**–**L**) Senegalese sole gonad. Results are represented as mean ± SE. Student-*t* test significant differences between ML and MD are identified with an asterisk, ANOVA II differences between seasons are identified with a cardinal (#). Statistical differences were considered when *p* < 0.05

In wild males, the expression of all genes except *hiomt1* was significantly affected by season, being all of them upregulated at OS (ANOVA II, *p* < 0.05) (Fig. 4 a and c–f). On the other hand, a significant effect of daytime was observed for *tph1a* and *aanat2*, which showed higher expression at night (MD) than during the day (ML), irrespective of the season (ANOVA II, *p* < 0.05) (Fig. 4 a and d). In F1 males, the expression of *tph1a* and *aanat1a* was also significantly affected by season, with higher expression levels found at OS (ANOVA II, *p* < 0.001) (Fig. 4 g and i).

As for *tph2*, *aanat1b*, and *mel1c*, no expression was detected in any of the analyzed tissues in the present study.

## Discussion

This study has measured the expression of genes coding for key enzymes from the melatonin biosynthetic pathway and melatonin receptors (*tph1a, aanat1a, aanat2, hiomt1, mel1*, and *mel2*) in the brain, eye and gonad of Senegalese sole (wild and F1) to determine whether these organs may be extra-pineal sites of melatonin production in this fish species. The obtained results demonstrated the expression of genes encoding enzymes involved in melatonin biosynthesis (*tph1a*, *aanat1a*, *aanat2*, *hiomt1*) and melatonin receptors (*mel1* and *mel2*) in all the organs investigated, suggesting their role as extra-pineal sites of melatonin production in Senegalese sole males. Previous studies performed in Senegalese sole by our group proved that melatonin was present in seminal plasma, contributing to the total antioxidant capacity of this fluid (Félix et al. 2023). The fact that the enzymes responsible for melatonin production and its receptors are simultaneously present in the testis may indicate a possible autocrine action of melatonin in this organ, as previously described in the retina of European seabass (Sauzet et al. 2008). The same mechanism was advanced by other authors in terms of melatonin production and usage in the ovary (Khan et al. 2016) and testis (Devi et al. 2021) of zebrafish. Similar to our findings, in zebrafish, the expression of *tph1*, *aanat1*, *aanat2*, and *asmt* proved the existence of enzymes involved in melatonin synthesis in both female and male reproductive structures (Devi et al. 2021), reinforcing the idea of these organs functioning as peripheral melatonin production sites in fish.

In our study, both seasonal and day/night variations were observed in the expression of several genes. In brain, for *tph1a*, a significant interaction season × daytime was found. Thus, during the BS, in wild males, the expression of *tph1a* was significantly higher at MD than at ML. However, in F1 males, the opposite pattern was observed. Also, no day/night differences in melatonin plasma levels were observed in this group, suggesting impaired nocturnal synthesis of melatonin during the reproductive season, which was not the case out of reproductive season. Nonetheless, the last enzyme acting in this process (HIOMT) had a similar pattern of gene (*hiomt1*) expression in fish from both origins, with higher expression at night during the BS, indicating that disruption primarily affects the early stages of melatonin synthesis in F1 sole, rather than the entire biosynthesis pathway. Additionally, although *aanat2* is generally associated with the daily regulation of melatonin production in the pineal organ (Falcón et al. 2010), in our experiment, no daily variations were observed in the expression of this gene, in opposition to previous investigations by other authors in Senegalese sole (Isorna et al. 2009). However, we hypothesize that these discrepancies may be partly explained by the fact that the whole brain was used in the present research to determine gene expression, instead of isolated pineal organs. Regarding the genes encoding the melatonin receptors, both *mel1* and *mel2* expressed in brain, eye and gonad of sole from both origins. However, seasonal and daily differences in the expression of these genes was both organ- and origin-dependent: in brain, no seasonal effects were found in wild or F1 fish, although day/night differences were observed for both genes in wild fish during BS (higher expression at night) and for *mel2* in F1 (higher expression during the day). Likewise, in Atlantic salmon (*Salmo salar*), both isoforms of melatonin receptors were expressed in different brain regions, but daily variations were only detected in the pituitary gland at the beginning of gonadal maturation (Ciani et al. 2019). Noteworthy, the different patterns of *mel2* expression between fish origins are in accordance with the lack of day/night variations in plasma melatonin levels in the F1 group during BS. This, together with the lowest levels of plasma melatonin at BS, suggests some dysregulation in the circadian system of F1 sole, since these results were not observed in wild type animals. The authors hypothesize that while melatonin appears to be more involved in preparing fish for reproduction than in regulating breeding season itself, the mechanisms underlying its impaired production in F1 remain unclear and require further research. In fact, the expression of melatonin receptors and the enzymes responsible for its synthesis were shown to be influenced by daytime and season (BS/OS), in all the tissues analyzed, as also described in photoperiodic mammals (Casao et al. 2010). These daily and seasonal variations in gene expression also changed according to the broodstock origin and are likely linked to the seasonal differences found in blood melatonin of F1 and wild animals.

In the eye, a strong effect of season was found for the expression of all the enzymes involved in melatonin synthesis in F1 males, with *tph1a* and *hiomt1* transcript levels being higher at OS. Yet, it is not clear if melatonin produced by the retina contributes to the higher blood melatonin concentrations found in F1 males out-of-season, or if it is metabolized locally (Falcón et al. 2007). Also, in the eye, *aanat1a* expression levels were higher during the BS for both fish origins. However, whereas wild fish displayed day/night differences (higher expression at night), F1 males did not. High expression of this enzyme in the fish eye had been previously documented in the retina of rainbow trout (Besseau et al. 2006), although in the retina of Senegalese sole previous studies have reported variations in the expression of *aanat1a* and *aanat1b* according to the developmental stage (Isorna et al. 2011). In fact, the subtype *aanat1b* was not detected in any tissue of the adult males used in the present study, which is in accordance with research reporting higher expression of this enzyme coinciding with metamorphosis, and suggesting an antagonistic relation with the melatoninergic system (Isorna et al. 2011). Regarding melatonin receptors, season affected the gene expression levels of *mel2* in the eye, although opposite patterns were found in wild (BS > OS) and F1 (BS < OS) sole. In turbot (*Scophthalmus maximus*), although no information about season variations were explored, *mel1* and *mel2* also expressed in the eye of both male and female, whereas *mel1c* only expressed in the skin (Zhao et al. 2021).

The expression of melatonin receptor genes *mel1* and *mel2* was determined in the brain, eye, and gonad. However, no expression of *mel1c* was found in any organ, in agreement with previous results in this species (Confente et al. 2010) and in Atlantic salmon (Ciani et al. 2019). In Senegalese sole, *mel1c* expression was found to be restricted to the muscle and skin, out of all central and peripheral tissue distribution analysis done (Confente et al. 2010), which also included retina and brain. Recent advances regarding the evolution of this receptor (MT3) in terms of structure and functions demonstrated higher relevance of this subunit among Cypriniformes (Li et al. 2021). However, *mel1c* expression has also been observed in different tissues of other marine fish species, such as the retina and brain of golden rabbitfish (*Siganus guttatus*) (Park et al. 2007), the pituitary and several peripheral tissues of orange-spotted grouper (*Epinephelus coioides*) (Chai et al. 2013), the skin of European seabass (Sauzet et al. 2008) and turbot (Zhao et al. 2021), and in unfertilized eggs of Senegalese sole (Lan-Chow-Wing et al. 2014). Nonetheless, some authors propose that MT3 should not be considered a receptor since it does not fulfil all the requirements (Acuña-Castroviejo et al. 2014): It demonstrated low affinity to 2-[^125^I]iodomelatonin and in the nanomolar range, is not coupled to G proteins, no signal transduction cascade has been identified yet, and it is not clear if has membrane binding sites (Slominski et al. 2012). Moreover, in mammals, some studies have showed its inability to respond to melatonin (Gautier et al. 2018).

In the testis, the expression of most enzymes involved in melatonin biosynthesis was affected by the season, being higher at OS than during BS (*tph1a* and *aanat1a* for both broodstocks and *aanat2* for wild males). Also, in wild fish, the expression of *tph1a* and *aanat2* was higher at night than during the day (OS), although these differences were abolished in the F1 group. Altogether, these results suggest that melatonin production also takes place in the testes of Senegalese sole, wherein it shows both seasonal and daily variations, likewise in other organs. Moreover, regarding melatonin receptors expression in testis, a seasonal effect was only observed in wild fish, in which both genes showed higher expression levels at OS. Previous studies in Senegalese sole demonstrated that the expression of melatonin receptors exhibits variations along the early development and metamorphosis (Lan-Chow-Wing et al. 2014), although in the present study only mature adult males were used. Therefore, further endocrinology research would be needed to determine whether these different patterns could be linked to reproductive impairment in F1 males. Recent studies with large yellow croaker (*Larimichthys crocea*) revealed that melatonin receptors MT1, MT2, and MT3 were present in fish testes and pituitary, but not in the female ovary, and the respective gene expression was higher in later sexual maturity stages (Liang et al. 2025). The melatonin receptors gene expression between males and females was not studied in our experiment but the expression of those genes in the testes of adult males was addressed. The present study proved the expression of two different melatonin receptor genes (*mel1* and *mel2*) in Senegalese sole testis, both of them displaying seasonal variations, only in wild males (OS > BS). This seasonal pattern matched that observed for the expression of *tph1a*, *aanat1a* and *aanat2* in this organ, which correspond to three key enzymes of the melatonin synthesis machinery, but had no relation with the melatonin concentration pattern in the blood plasma (no seasonal differences found in wild males). This could be indicative of a gonadal melatonin regulation independent of blood melatonin, as previously advanced by our group (Félix et al. 2023). Moreover, in F1 males, the observed pattern was different than in wild animals: Seasonal differences were observed in blood melatonin (OS > BS), and no seasonal differences were found in the expression of *aanta2*, *mel1*, and *mel2*. The fact that, in the testis, the expression of enzymes and receptors was higher OS in the wild fish, is probably related to the reproductive strategy adopted by the species. Senegalese sole is a long-day breeder, thus the increasing light period from winter onwards is an inducer of gonadal maturation (Falcón et al. 2007). This environmental information is transduced to the organism through the production of melatonin (Falcón et al. 2010), which seems to be increased in the Senegalese sole testes OS, preparing the fish for the BS. However, even prioritizing the selection of fish with similar sizes, maturity stages, rearing conditions and water temperature, the influence of genetic variability cannot be rolled out.

In short, the present study brings new information related to the tissue distribution of AANAT isoforms in Senegalese sole and, consequently, its melatonin production system. Literature often refers to *aanat1a* as a retinal gene isoform (Klein 2007); however, in the present experiment, it expressed in all the tissues analyzed (brain, eye, and testis). Similar results were observed in European seabass, in which *aanat1a* expression was found in gonad, intestine, liver, and muscle (Paulin et al. 2015). Similarly, *aanat2* is normally expressed in the pineal organ but our study demonstrated to be also present in the testis of Senegalese sole. Also, in *Catla catla* females, *tph1*, *hiomt*, *annat1*, and *aanat2* expressed in several tissues, including the brain, pineal organ, retina, muscle, heart, gut, kidney, liver, and ovary (Rajiv et al. 2016). Altogether, these gene expression results suggest a wider tissue distribution of these enzymes in fish species, which opens the door to future research on their putative physiological roles in extra-pineal organs. Similarly, in the present study, the cellular location of melatonin receptors was not investigated. However, in mammalian studies with wild roe deer (*Capreolus capreolus*) testes, it was proven that MT1 and MT2 were present not only in Leydig, Sertoli, and germ cells but also in the epithelium cells along the epididymis, influencing steroidogenesis, spermatogenesis, and sperm maturation processes (Koziol et al. 2020). Thus, there is space to deepen the research and reach a cellular level understanding by exploring which type of cells are responsible for this melatonin production in the several studied tissues.

## Conclusions

Our findings revealed that genes *tph1a*, *aanat1a*, *aanat2*, and *hiomt1*, key factors for melatonin biosynthesis, expressed in the testis of Senegalese sole males, as well as the melatonin receptors genes *mel1* and *mel2*, suggesting that the respective enzymes are present in testis, and thus, the male gonads are an extra-pineal production site of melatonin. Moreover, in fish from both broodstocks (F1 and wild males) and in all tissues (brain, eye and gonads), the expression of those enzymes and receptors in the brain-gonadal axis presented daily and/or seasonal variations. Nevertheless, compared to the wild males, the F1 group showed different expression patterns for some genes in all the analyzed tissues. Also, the expression of the gene *mel1c* (MT3 receptor) was not detected in any tissue or fish group.

## Data Availability

No datasets were generated or analysed during the current study.
